# Endoscopic ultrasound-guided esophageal hematoma drainage after radiofrequency ablation for atrial fibrillation

**DOI:** 10.1055/a-2658-0422

**Published:** 2025-08-01

**Authors:** Mustafa Suveran, Can Boynukara, Duhan F. Bayrak, Sena Sert Sekerci, Gurhan Sisman

**Affiliations:** 1 713016 Department of Gastroenterology and Hepatology, Acibadem Altunizade Hospital, Istanbul, Turkey; 2 Department of Internal Medicine, Acibadem Mehmet Ali Aydınlar University, Istanbul, Turkey; 3 713016 Department of Cardiology, Acibadem Altunizade Hospital, Istanbul, Turkey; 4 Department of Gastroenterology and Hepatology, Acibadem Mehmet Ali Aydınlar University, Istanbul, Turkey

Transesophageal radiofrequency ablation (RFA) for atrial fibrillation may cause esophageal injuries, including hematomas, which are rare but potentially serious 1 . Endoscopic ultrasonography (EUS) is a valuable tool for both diagnosis and minimally invasive management of such complications, enabling precise targeting and drainage 2 .

A 67-year-old woman developed dysphagia, odynophagia, epigastric pain, and inability to tolerate oral intake after transesophageal RFA. Gastroscopy (Evis Exera III GIF H190; Olympus, Japan) revealed a lesion obstructing the esophageal lumen at 18 cm ( Video 1 ). EUS (GF-UCT linear ultrasound endoscope; Olympus, Japan) confirmed a submucosal hematoma, and thoracic computed tomography (CT) showed a 4-cm hematoma in the mid-esophagus. EUS-guided drainage was performed. The procedure involved puncturing the hematoma with a 19-gauge needle (Expect; Boston Scientific, United States) and placing a 0.035-inch guidewire (VisiGlide; Olympus, Japan) under fluoroscopic guidance. The tract was dilated using a 6-Fr cystotome (Cystotome; Endo-Flex, Germany), and a 7-Fr, 5-cm plastic pigtail stent (Bile Duct Stents; Endo-Flex, Germany) was inserted into the hematoma cavity. Enteral nutrition was started via a nasogastric tube. One week later, CT showed the hematoma size reduced to 2.5cm. The stent was removed 1 month later, with a follow-up endoscopy revealing mucosal ulcerations. The esophageal mucosa was normal at 6 months, and the lumen remained patent.

Endoscopic ultrasound-guided esophageal hematoma drainage after radiofrequency ablation for atrial fibrillation.Video 1

This case demonstrates the efficacy of EUS-guided drainage for esophageal hematomas occurring after RFA. Early intervention prevents progression to perforation or fistula, which can be fatal 3 . EUS offers several advantages: it enables real-time visualization, precise targeting, and safe decompression, minimizing risks associated with surgical or blind procedures. Its minimally invasive nature allows faster recovery and mucosal healing, as seen in this patient. Given the increasing use of transesophageal ablation techniques, EUS-guided management should be considered a valuable option in similar cases 1 2 . To our knowledge, this case is the first such reported in the English-language literature.

Endoscopy_UCTN_Code_TTT_1AS_2AC

## Notes

Endoscopy E-Videos https://eref.thieme.de/e-videos
*E-Videos* is an open access online section of the journal *Endoscopy* , reporting on interesting cases and new techniques in gastroenterological endoscopy. All papers include a high-quality video and are published with a Creative Commons CC-BY license. Endoscopy E-Videos qualify for HINARI discounts and waivers and eligibility is automatically checked during the submission process. We grant 100% waivers to articles whose corresponding authors are based in Group A countries and 50% waivers to those who are based in Group B countries as classified by Research4Life (see: https://www.research4life.org/access/eligibility/ ). This section has its own submission website at https://mc.manuscriptcentral.com/e-videos .
